# Incidentally Identified Pulmonary AVM: An Unusual Cause for Stroke in a Young Patient

**DOI:** 10.1155/2020/1203945

**Published:** 2020-08-13

**Authors:** Vinod Kalapurackal Mathai, Dale Sebire, Scarlett Bowen

**Affiliations:** The Royal Hobart Hospital, Hobart, Australia

## Abstract

Pulmonary arteriovenous malformation is an uncommon though important potential source for embolic right-to-left extracardiac shunt as a cause for both silent and clinically evident stroke. We present this case to highlight this pathology as a cause for stroke, the importance of treating this malformation, even if incidentally identified, and finally the role of echocardiography in screening for extracardiac shunt and indicating those patients that may benefit from further investigations looking for extracardiac shunt.

## 1. Case

We present the case of a previously well 42-year-old lady who presented recently to a tertiary public hospital with right-sided weakness suggestive of stroke.

Past medical history was limited to depression treated with venlafaxine. At presentation, this patient had described a fall the previous day following acute onset right leg and arm weakness. The weakness had resolved after several hours, and her gait normalized. She had presented to the emergency department the following morning. It was also reported that she had experienced an episode of expressive dysphasia three weeks prior which had settled after forty-eight hours. She was a lifelong nonsmoker, had never been diagnosed with hypertension, dyslipidemia, or diabetes, and had no personal or family history of young onset stroke or venous thromboembolism.

**Figure 1:**
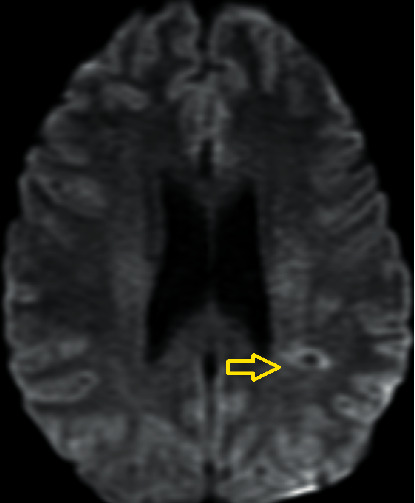
Diffusion restricted image of the left posterior parietal lobe.

**Figure 2:**
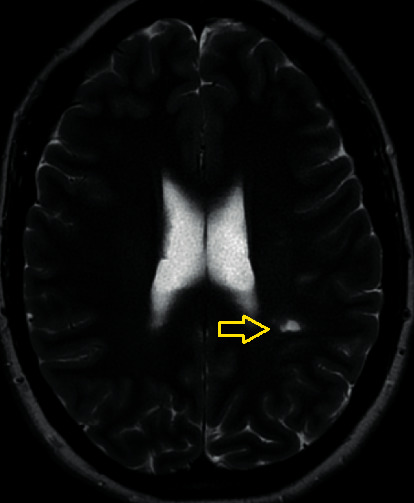
T2 weighted image of left posterior parietal lesion.

**Figure 3:**
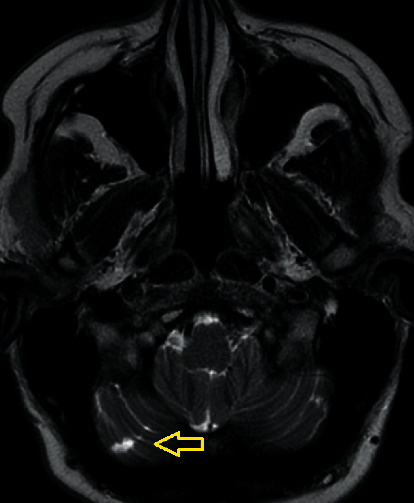
Right cerebellar lesion.

This lady was admitted for investigation including MRI brain. This confirmed a focal area of subacute left MCA territory minimal restricted diffusion (Figure 1) and an area of increased T2 signal intensity in the left posterior parietal lobe (Figure 2). It was thought that this finding reflected a stroke with the expressive dysphasia two weeks earlier. There were no hyperacute findings on the MRI. Interestingly, MRI brain also revealed evidence of probable chronic infarct in the right cerebellum (Figure 3).

A subacute ischaemic stroke was diagnosed based on imaging with probable TIA on the day prior. Further investigations were undertaken. Thrombophilia screen for hereditary and acquired prothrombotic conditions was completed in addition to autoimmune and vasculitis serology. HbA1C, syphilis serology, HIV, and Hep B and C testing were all unremarkable.

**Figure 4:**
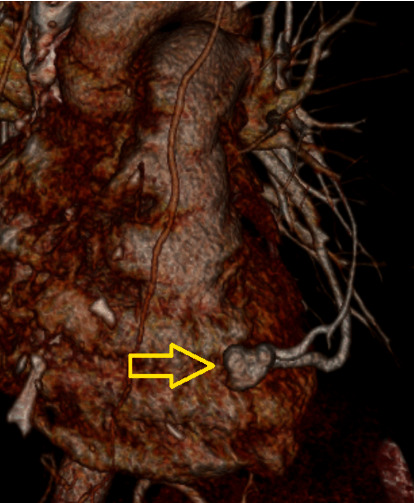
CTPA reconstruction of left lower-lobe PAVM from 2011.

The case was tabled for discussion at the departmental weekly neuroradiology meeting. The MRI brain along with previous imaging was reviewed. Attention was also given to a computed tomography pulmonary angiogram (CTPA) that had been undertaken in 2011 by way of investigation for pleuritic chest pain. Pulmonary embolism was excluded; however, noted was a pulmonary arteriovenous malformation (PAVM) in the anterior left lower-lobe (Figure 4). At the time of CTPA in 2011, the significance of this incidental malformation had not been appreciated by the emergency department staff, and the patient had not been referred for management of this potentially dangerous anomaly. There had been no suggestion of a previous stroke-like syndrome to raise the concern that the PAVM had been symptomatic. Often a PAVM will be evident on plain chest X-ray, though, in this lady, it was not evident (obscured by the cardiac shadow). The sensitivity for chest X-ray identifying PAVM is around 60% [1].

## 2. Impression

In the absence of typical risk factors for a stroke, the patient's young age, imaging evidence of both old and subacute infarcts in more than one vascular territory, and the failure to find an alternate medical cause for stroke, it was determined that the PAVM was a likely conduit for right-to-left embolic thrombus, and treatment was planned. There was no FHx of hereditary haemorrhagic telangiectasia (HTT) and no medical history of conditions that may predispose to the development of a PAVM. This patient underwent urgent digital subtraction angiography and coil embolization and was discharged soon after without neurological deficit.

## 3. Discussion

The incidence of PAVM is known to be very low based on the limited epidemiological data available. An autopsy study in 1953 revealed just three cases of PAVM from 15,000 autopsies [2]. A subsequent study looked at the incidence of PAVM in Japan with asymptomatic patients undergoing low-dose CTPA screening from 2001 to 2007. Eight cases of PAVM in 21,235 screened patients were detected creating an incidence of around to 1 in 2500 persons [3]. It is not clear, however, how frequently PAVM can be found in cases of cryptogenic stroke largely because screening with CTPA is not common practise and it may not always be considered with echocardiography.

It is widely recognised that the majority (around 70%) of PAVMs identified will be associated with HHT [4]. There are a number of acquired conditions that may less commonly result in PAVM including, but not limited to, hepatic cirrhosis, penetrating chest trauma, and thyroid carcinoma [5].

The finding of a PAVM is a concern and particularly in the setting of acute stroke. It has been shown that the prevalence of imaging confirmed ischaemic stroke lesions in a patient with PAVM is 32% and 60% if multiple PAVM [6]. Asymptomatic stroke is also significant with one small study showing 15 out of 21 patients (68%) with HHT and PVAMs also showing MRI brain evidence of silent ischaemic stroke with a median age of just 43 [7].

Given the very low incidence of PAVM, how best can this possibility be excluded in cases of cryptogenic stroke without subjecting a great many patients to the radiation associated with CTPA? Identifying such a structural abnormality is critical to prevent stroke recurrence. Echocardiogram with bubble contrast study likely represents the best opportunity to identify such abnormalities. Prior screening studies have shown a positive transthoracic contrast echocardiography (bubble study) to have a strong positive predictive value for detecting PAVMs especially if higher grade left ventricular opacification is seen [8]. A further study compared this modality with computed tomography in paediatric patients with genetic testing supportive of a diagnosis of HHT and found that the sensitivity and specificity of contrast echocardiography for the detection of PAVM's was 100% and 95%, respectively [9].

It has been established that the time or number cardiac cycles, postinjection, taken for bubbles to become apparent in the LA and LV is prolonged when PAVM is compared to the more typical direct interatrial shunt (66 frames versus 21 frames) [10] This might equate to 1–3 cardiac cycles for a typical intracardiac right-to-left shunt, 3–5 being equivocal, and beyond 5 cycles is highly suggestive of an extracardiac source of right-to-left shunt. If echocardiography is suggestive of extracardiac shunt, then moving to CTA or directly to DSA and embolization would be indicated.

## 4. Recommendations

Firstly, it is critical that the importance of a finding of PAVM needs to be understood even if detected incidentally. Computed tomography of the chest is performed routinely across the world for a great many indications, and being aware of the standard management of vascular malformations is clearly necessary. In this case, it was fortuitous that the patient's stroke symptoms had been mild with full resolution, and ultimately definitive coiling was provided. Timely referral, however, may have prevented what could have been a very deleterious outcome.

Secondly, highlighting the possibility of extracardiac sources of emboli as a source of cryptogenic stroke is very important, particularly among general and emergency physicians, neurologists, cardiologists, and echocardiographers. Contrast echocardiography should be performed with due attention particularly to the presence of a right-to-left shunt, and critically the timing of the appearance of contrast bubbles in the left atrium and ventricle should be documented. If more than 3 cardiac cycles elapse, and certainly if that number gets beyond 5 prior to the appearance of left atrial bubbles, then consideration of imaging to detect an extracardiac source of shunt is suggested.

## Data Availability

The data used to support the findings of this study are available from the corresponding author upon request.

## References

[1] Andersen P., Elle B., Jacobson J., Kjeldsen A. D., Oxhoj H., Vase P. (1999). Pulmonary arteriovenous malformations: screening procedures and pulmonary angiography in patients with hereditary hemorrhagic telangiectasia. *Chest*.

[2] Cooley R. N., Sloan R. D. (1953). Congenital pulmonary arteriovenous aneurysm. *The American Journal of Roentgenology Radium Therapy and Nuclear Medicine*.

[3] Bando M., Chonan T., Endo K. (2012). Prevalence of pulmonary arteriovenous malformations as estimated by low-dose thoracic CT screening. *Internal Medicine*.

[4] Gossage J. R., Kanj G. (1998). Pulmonary arteriovenous malformations. *American Journal of Respiratory and Critical Care Medicine*.

[5] Bhalla S., Cummings W. (2015). Pulmonary vascular diseases. *Clinics in Chest Medicine*.

[6] Fayad P., Hashimoto M., Henderson K. (2000). Pulmonary arteriovenous malformations: cerebral ischemia and neurologic manifestations. *Neurology*.

[7] Cottin V., Dupuis-Girod S., Shovlin C. (2017). The lung in hereditary hemorrhagic telangiectasia. *Respiration*.

[8] Chan R., Chow C., Cohen J., Faughnan M., Zukotynski K. (2007). Contrast echocardiography grading predicts pulmonary arteriovenous malformations on CT. *Chest*.

[9] Blivet S., Chinet T., Duborg O. (2015). Reliability of contrast echocardiography to rule out pulmonary arteriovenous malformations and avoid CT irradiation in pediatric patients with hereditary hemorrhagic telangiectasia. *Echocardiography*.

[10] Barzilai B., Waggoner A. D., Spessert C., Picus D., Goodenberger D. (1991). Two-dimensional contrast echocardiography in the detection and follow-up of congenital pulmonary arteriovenous malformations. *The American Journal of Cardiology*.

